# Parental exposures to occupational asthmagens and risk of autism spectrum disorder in a Danish population-based case-control study

**DOI:** 10.1186/s12940-017-0230-8

**Published:** 2017-03-31

**Authors:** Alison B. Singer, Igor Burstyn, Malene Thygesen, Preben Bo Mortensen, M. Daniele Fallin, Diana E. Schendel

**Affiliations:** 1 Department of Epidemiology and Wendy Klag Center for Autism and Developmental Disabilities, Johns Hopkins University Bloomberg School of Public Health, 615 N. Wolfe St, Baltimore, MD 21205 USA; 2 Centre for Integrated Register-based Research, Aarhus University, Aarhus, Denmark; 3 Department of Epidemiology, University of North Carolina at Chapel Hill, CB #7435, Chapel Hill, NC 27599 USA; 4 Department of Environmental and Occupational Health, Department of Epidemiology and Biostatistics, the A.J. Drexel Autism Institute, Drexel University Dornsife School of Public Health, 3215 Market Street, Philadelphia, PA 19104 USA; 5 Department of Economics and Business, National Centre for Register-based Research, Aarhus University, Fuglesangs Allé 4, Building 2631, DK-8210 Aarhus V, Denmark; 6 Lundbeck Foundation Initiative for Integrative Psychiatric Research, iPSYCH, Aarhus, Denmark; 7 Department of Mental Health, Johns Hopkins University Bloomberg School of Public Health, 624 N. Broadway, Baltimore, MD 21205 USA; 8 Department of Public Health, Section for Epidemiology, Aarhus University, Bartholins Allé 2, Building 1260, DK-8000 Aarhus C, Denmark

**Keywords:** Epidemiology, Neurodevelopment, Prenatal exposure, Occupational asthma, Autism

## Abstract

**Background:**

Environmental exposures and immune conditions during pregnancy could influence development of autism spectrum disorder (ASD) in offspring. However, few studies have examined immune-triggering exposures in relation to ASD. We evaluated the association between parental workplace exposures to risk factors for asthma (“asthmagens”) and ASD.

**Methods:**

We conducted a population-based case-control study in the Danish population using register linkage. Our study population consisted of 11,869 ASD cases and 48,046 controls born from 1993 through 2007. Cases were identified by ICD-10 codes in the Danish Psychiatric Central Register. ASD cases and controls were linked to parental Danish International Standard Classification of Occupations (DISCO-88) job codes. Parental occupational asthmagen exposure was estimated by linking DISCO-88 codes to an asthma-specific job-exposure matrix.

**Results:**

Our maternal analyses included 6706 case mothers and 29,359 control mothers employed during the pregnancy period. We found a weak inverse association between ASD and any maternal occupational asthmagen exposure, adjusting for sociodemographic covariates (adjusted OR: 0.92, 95% CI: 0.86–0.99). In adjusted analyses, including 7647 cases and 31,947 controls with employed fathers, paternal occupational asthmagen exposure was not associated with ASD (adjusted OR: 0.98, 95% CI: 0.92–1.05).

**Conclusions:**

We found a weak inverse association between maternal occupational asthmagen exposure and ASD, and a null association between paternal occupational exposure and ASD. We suggest that unmeasured confounding negatively biased the estimate, but that this unmeasured confounding is likely not strong enough to bring the effect above the null. Overall, our results were consistent with no positive association between parental asthmagen exposure and ASD in the children.

**Electronic supplementary material:**

The online version of this article (doi:10.1186/s12940-017-0230-8) contains supplementary material, which is available to authorized users.

## Background

Autism spectrum disorder (ASD) is a diverse neurodevelopmental condition characterized by repetitive or stereotypic behaviors and impairments in social communication and interactions. Studies suggest a possible link between maternal immune conditions and ASD [1–4], though links between specific maternal immune conditions have been inconsistent [1, 5, 6]. Many agents (“asthmagens”) have been linked to triggering or exacerbating asthma in the workplace, including animal antigens, pharmaceutical drugs, reactive dyes, cleaning products, metals, and wood dust [7]. People working in a variety of jobs, including nurses, cleaners, carpenters, crop and animal producers, bakers, and hairdressers may be exposed to workplace asthmagens [8].

Exposure to asthmagenic agents may trigger asthma by causing an immune response or irritation that results in airway damage and inflammation. We hypothesized that in some individuals asthmagen exposure could alter the maternal in utero environment and increase risk for ASD in the offspring by (a) triggering an immune response or by (b) decreasing oxygen supply to the fetus. Links between ASD and ambient air pollutant exposures to certain types of asthmagens (acetaldehyde, formaldehyde, styrene, and metals) have been reported, though associations are not observed in all studies [9–12]. In contrast to air pollutant exposures, occupational exposures can be more intense and estimated based on individual employment. McCanlies et al. [13] linked ASD with self-report of occupational asphalt and solvent exposure, and industrial hygiene assessment of lacquer, xylene, and varnish exposure. Windham et al. [14] found an association between ASD and any maternal chemical occupational exposure, as well as exhaust/combustion products from engines and disinfectants, specifically. Another case-control study comparing children with ASD to children with other developmental conditions reported associations between maternal and paternal occupational solvent use and ASD [15]. In Singer et al. [16], we examined the association between maternal exposure to occupational asthmagens and ASD in the Study to Early Development (SEED), a U.S. case-control study. In the SEED study, our findings were consistent with a null association between maternal asthmagen exposure and ASD (aOR: 1.39, 95% CI: 0.96–2.02). However, limitations of this analysis included a small sample size and concern over potential selection bias.

Here, we conducted a large study examining occupational exposure to asthmagens in relation to ASD in a population-based case control study using data from Danish Registers. Since fathers may bring asthmagens home from the workplace, indirectly exposing mothers to paternal occupational asthmagens [17, 18], we examined the association between both maternal and paternal workplace exposure to asthmagens and ASD. Different forms of ASD may have different etiologic pathways, so we examined the association between parental occupational asthmagen exposure and both strict childhood autistic disorder and ASD with intellectual disability. We also evaluated whether history of asthma modified the association between asthmagen exposure and ASD because asthma history may impact maternal immune response or exposure. Finally, since the prevalence of ASD differs by child sex, we also examined the association between asthmagens and ASD separately for male and female children.

## Methods

### Study design

All live-born children and new Danish residents are assigned a unique personal identification number that links information across different national registers. We linked children to parents using the Danish Civil Registration System [19]. We linked to the Danish Psychiatric Central Register (DPCR) [20] for information on psychiatric diagnoses, the Medical Birth Registry [21] for birth-related covariates, the National Patient Register [22] for medical diagnoses, the Danish National Prescription Registry [23] for dispensed prescription medications, and Statistics Denmark registers for information on employment, education, and finance [24, 25].

### Selection of eligible participants

We identified all children in the Danish Civil Registration System born between January 1, 1993 and December 31, 2007 (*n* = 1,099,463). We excluded children who could not be linked back to the mother through the Danish Civil Registration System (*n* = 15,404); children who were not born in Denmark, with unknown birthplace, or with no match in the Medical Birth Registry (*n* = 88,908); children missing gestational age or with gestational ages at birth of <23 weeks or >43 weeks (*n* = 8945); and children from multiple births or missing information on multiplicity (*n* = 38,638). Thus, we selected our sample from 947,568 individuals meeting the above study inclusion criteria. The study was approved by the Danish Data Protection Agency (J. nrs. 2013-231-0054 and 2014-231-0088).

### Case and control selection

The Danish Psychiatric Central Register (DPCR) [19] contains diagnoses from every inpatient psychiatric admission from 1970 to present, and from every outpatient contact or treatment from January 1, 1995 [20]. International Classification of Diseases, 10^th^ Revision (ICD-10) classification has been used for reporting of psychiatric diagnoses since January 1, 1994. In Denmark, children with a suspected ASD are referred to a psychiatric ward where they receive a diagnostic evaluation and obtain a diagnosis from a psychiatrist that is entered into the DPCR. Cases had a reported diagnosis of ASD (ICD-10 codes: F84.0, F84.1, F84.5, F84.8 and F84.9) from January 1, 1995 into April 2013. Due to time lags in reporting diagnoses to the DPCR, the diagnosis data are considered complete through the end of 2012. For every case (*n* = 12,500), we randomly sampled four controls without an ASD diagnosis and meeting the study inclusion criteria. The study sample was further restricted to only include the oldest child for each mother and to exclude those lost to follow-up before age one, children with inconsistent maternal identification numbers across registers, and children with likely erroneous birth weights for gestational age (defined as birth weight more than six standard deviations from the mean sex-specific birth weight for each gestational age week calculated from Danish-born singletons from 1980 to 2007). The final study sample consisted of 11,869 ASD cases and 48,046 controls. We defined strict childhood autistic disorder as any case having at least one F84.0 diagnosis and ASD with intellectual disability as any case with a F70, F71, F72, F73, F78, or F79 diagnosis in the DPCR.

### Employment status definition

Since the occupational register is updated once every year, we set a rule for determining which year of job information to use to estimate prenatal exposure. For children born from January through May, we used parental job information from the year prior to the child’s birth year. For children born from June through December, we used job information from the year of birth. Hereafter, we refer to the year of job information as the “occupational year.” Employment status is determined each year at the end of November, so we defined a parent as employed if s/he was employed in the November prior to the occupational year according to the Integrated Database for Market Research (IDA).

### Occupational asthmagen exposure assessment

We estimated parental occupational asthmagen exposure during the pregnancy period by linking job codes from the occupational year in the Employment Classification Module (AKM) [24] to an asthma-specific job exposure matrix (JEM) developed by Kennedy et al [8]. Job codes are in the format of the Danish International Standard Classification of Occupations (DISCO-88), the Danish version of the International Standard Classification of Occupations (ISCO-88). During the time period of this study, the DISCO-88 codes represent the person’s primary (i.e. highest salary) job.

The asthma-specific JEM matches ISCO-88 codes to categories of occupational asthmagen exposures [8]. Each job code in the asthma-specific JEM is given a classification of “yes” or “no” as to whether or not an individual employed in the job has a high probability of being exposed to a specific asthmagen. The JEM contains four different subgroups of agents that would put an individual at risk of developing asthma: (1) high molecular weight agents, (2) low molecular weight agents, (3) mixed environments with multiple agents that can be a combination of HMW and LMW compounds, and (4) irritant peaks, as well as compounds or mixtures that belong to each category. We assigned exposure on the basis of harmonizing ISCO-88 and DISCO-88 codes.

The developers of the asthma-specific JEM suggest completing a two-step verification step in order to improve exposure classification where (1) ISCO-88 codes are checked and (2) asthmagen exposure classifications are checked and potentially reclassified based on industry and job tasks. We did not have job task information, but where possible we used industrial codes to re-classify occupational asthmagen exposures according to suggestions in the JEM. We coded carpenters and joiners (ISCO-88 code 7124) as exposed to wood dust. Wood species used in Danish furniture factories have been associated with respiratory symptoms [26]. These assessments were made without consideration of case or control status.

### Creation of other covariates

The variables for index child’s sex, child’s birth year, maternal and paternal age at child’s birth, parity, urbanicity, and parental immigrant status were derived from the Danish Civil Registration System. Age variables were calculated using the 15^th^ day of the month and year of birth. Maternal smoking at any point during the pregnancy was obtained from the Medical Birth Registry. Parental income and education were derived from the Statistics Denmark personal finance and education databases. To calculate parental income, we summed maternal and paternal income. In situations where maternal income was missing, we assumed that the parental income was the paternal income, and vice versa. Highest level of education was defined as the highest level of education of either the mother or the father. In cases where the education level of one of the parents was missing, we used the education level of the other parent. History of parental psychiatric diagnosis was defined as any ICD-8 diagnosis in the range of 290-315 or any ICD-10 diagnosis in the F group in the DPCR in either parent prior to the child’s delivery. We identified maternal or paternal asthma diagnoses any time prior to the child’s delivery by an ICD-8 diagnosis of 493 or an ICD-10 diagnosis of J45 or J46 in the Danish National Patient Register, which contains medical diagnoses by specialists. We identified maternal prescription for an asthma medication by Anatomical Therapeutic Chemical (ATC) Classification System codes of R03A (inhaled adrenergics), R03B (other inhaled drugs for obstructive airway disease), R03C (systemic adrenergics), or R03D (other systemic drugs for obstructive airway disease) in the Danish National Prescription Registry.

### Statistical analysis

We restricted the maternal analyses to employed mothers and paternal analyses to employed fathers. We also excluded parents for whom we could not estimate occupational asthmagen exposure because the DISCO-88 code was unknown or could not be cross-linked to an ISCO-88 code in the JEM. Among employed parents, we compared parents for whom we could estimate asthmagen exposure to those missing asthmagen exposure information. We restricted our analyses to parent-child pairs with complete covariate information.

We tabulated and calculated percentages of occupational asthmagen unexposed and exposed parents by various sociodemographic characteristics. We used logistic regression to model the association between parental exposure to any occupational asthmagen and ASD, adjusting for previously identified possible confounders including: child’s year of birth (dummy categories for year), child’s sex, maternal age at birth (continuous), paternal age at birth (continuous), parity (one, two, greater than 2), total parental income during the occupational year (<200,000 DKK, 200,000-399,999 DKK, 400,000 DKK-599,999 DKK, ≥600,000 DKK), highest parental education as of the occupational year (basic school, upper secondary school, vocational school, higher education), psychiatric diagnosis for either parent prior to the child’s birth (yes, no), maternal smoking at some point in the pregnancy (yes, no), urbanicity of birthplace (capital, capital suburb, provincial city, provincial town, rural area), and whether or not the parent was born in Denmark (yes, no). Maternal and paternal analyses were performed separately. Logistic regression was also used to model the association between specific occupational asthmagens and ASD. We conducted sensitivity analyses where we additionally adjusted for parental asthma diagnosis prior to the delivery of the child. We also examined the association between asthmagen exposure and subgroups of ASD, comparing childhood autism cases to controls and ASD with intellectual disability to controls.

The association between parental occupational asthmagen exposure and ASD may differ depending on whether or not a parent has a history of asthma. As such, we also fit logistic regression models with an interaction term for asthmagen exposure and history of asthma diagnosis prior to the child’s birth, and estimated the independent and joint effects of occupational asthmagen exposure and asthma. We conducted an additional effect modification analysis where we broadened the definition of maternal asthma history to include either an asthma diagnosis or prescription for an asthma medication prior to delivery of the child. We also fit logistic regression models with an interaction terms between any asthmagen exposure and child’s sex. We used the coefficients from these models to calculate sex-specific associations between asthmagen exposures and ASD. Logistic regression models were fit with PROC LOGISTIC using SAS software, Version 9.4 (SAS Institute, Cary, NC).

We assessed sensitivity to an unobserved confounder using the method described in Lin et al [27]. We were particularly concerned that an unmeasured confounder, such as a health factor (e.g. undocumented allergy or respiratory disease), may lead to avoidance of asthmagen exposed jobs and may also be a risk factor for ASD. Thus, we assumed that unmeasured confounder, U, is positively associated with ASD and inversely associated with maternal exposure. We assumed that the underlying prevalence of the unmeasured confounder among the unexposed was 30%. We then calculated corrected adjusted odds ratios for the association between maternal occupational asthmagen exposure and ASD, using the observed adjusted odds ratio and 95% confidence intervals and assuming different plausible values for the association between the unmeasured confounder and ASD (ORyu), and for the association between the unmeasured confounder and exposure (ORxu).

## Results

Of the 11,869 cases and 48,046 controls, mothers of 8416 (70.9%) cases and 36,090 (75.1%) controls were classified as employed. Fathers of 9707 (81.8%) cases and 40,619 (84.5%) controls were employed. We excluded 6732 children (1327 cases and 5405 controls) from maternal and 8993 children (1683 cases and 7310 controls) from paternal analyses because we could not estimate parental asthmagen exposure. Among employed parents, the parents missing exposure information tended to be lower socioeconomic status, younger, and more likely to be born outside of Denmark. Mothers missing asthmagen exposure information were more likely to smoke during pregnancy. Children of those missing asthmagen exposure information were more likely to born in later birth years. Of those remaining after these exclusions, some covariate information was missing for 383 ASD cases and 1326 controls in the maternal sample and 377 ASD cases and 1362 controls in the paternal sample. Thus, maternal analyses ultimately included 6706 cases and 29,359 controls, and paternal analyses included 7647 cases and 31,947 controls.

**Fig. 1:**
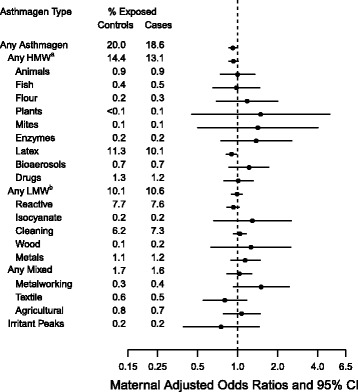
Adjusted odds ratios and 95% confidence intervals for maternal occupational asthmagen exposure and ASD. Maternal model is adjusted for child’s year of birth, child’s sex, maternal age at birth, paternal age of birth, total income of parents, parity, highest parental education, history of parental psychiatric diagnosis prior to child’s date of birth, urbanicity of birth place, maternal immigrant status, and maternal smoking (6706 cases and 29,359 controls). ^a^HMW = High Molecular Weight. ^b^LMW = Low Molecular Weight

**Fig. 2:**
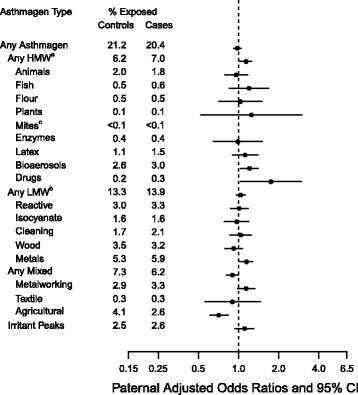
Adjusted odds ratios and 95% confidence intervals for paternal occupational asthmagen exposure and ASD. Paternal model is adjusted for child’s year of birth, child’s sex, maternal age at birth, paternal age of birth, total income of parents, parity, highest parental education, history of parental psychiatric diagnosis prior to child’s date of birth, urbanicity of birth place, paternal immigrant status, and maternal smoking (7647 cases and 31,947 controls). ^a^HMW = High Molecular Weight. ^b^LMW = Low Molecular Weight. ^c^Paternal mites odds ratio could not be estimated

In the occupational year, 19.8% of mothers and 21.1% of fathers were exposed to occupational asthmagens (Table 1). In table 1, we present parent and child characteristics by exposure to maternal and paternal asthmagens. In supplemental analyses, we also stratify these tabulations by case-control status (Additional file 1: Table S1, Table S2). The most prevalent jobs with maternal asthmagen exposure for cases and controls combined were nursing associate professionals (29.5%), institution-based personal care workers (24.0%), cleaning staff in non-domestic settings (8.0%), and hairdressers and beauticians (5.9%) with the most common exposures including latex, reactive agents, and cleaning products (Fig. 1, Additional file 1: Table S3). The most prevalent paternal jobs with asthmagen exposures were carpenters and joiners (16.5%), market-oriented crop and animal producers (12.3%), meat and fish processing machine operators (7.4%), and machine-tool setters and setter-operators (6.5%). The most common paternal exposures were metals, mixed environment agricultural antigens, wood dusts, reactive agents, metal working fluids, and bioaerosols (Fig. 2, Additional file 1: Table S4).

**Table 1:** Parent and child characteristics by occupational asthmagen exposure

Characteristic	Maternal Exposed^a^			Paternal Exposed^b^		
	No	Yes		No	Yes	
	N	N	%^c^	N	N	%^c^
Overall	28,942	7123	19.8	31,258	8336	21.1
Child’s Sex						
Male	16,294	3928	19.4	17,666	4614	20.7
Female	12,648	3195	20.2	13,592	3722	21.5
Child’s Year of Birth						
1993–1997	9613	3038	24.0	10,837	3492	24.4
1998–2002	10,462	2185	17.3	11,089	2838	20.4
2003–2007	8867	1900	17.6	9332	2006	17.7
Parity						
1	13,992	3191	18.6	14,264	3570	20.0
2	10,696	2613	19.6	11,982	3070	20.4
≥ 3	4254	1319	23.7	5012	1696	25.3
Mother’s Age at Child’s Birth						
≤ 25 years	4235	1180	21.8	5017	1970	28.2
26–30 years	11,748	2954	20.1	12,594	3335	20.9
31–35 years	9497	2232	19.0	10,099	2202	17.9
≥ 36 years	3462	757	17.9	3548	829	18.9
Father’s Age at Child’s Birth						
≤ 25 years	2130	562	20.9	2406	978	28.9
26–30 years	9085	2320	20.3	9576	2784	22.5
31–35 years	10,552	2531	19.3	11,227	2770	19.8
≥ 36 years	7175	1710	19.2	8049	1804	18.3
Total Parental Income						
< 200,000 DKK	539	121	18.3	785	216	21.6
200,000–399,999 DKK	7188	2227	23.7	8479	3217	27.5
400,000–599,999 DKK	14,078	3460	19.7	14,461	3888	21.2
≥ 600,000 DKK	7137	1315	15.6	7533	1015	11.9
Highest Parental Education						
Basic School	1506	580	27.8	2237	845	27.4
Upper Secondary School	1394	235	14.4	1615	302	15.8
Vocational School	12,411	3042	19.7	12,423	4909	28.3
Higher Education	13,631	3266	19.3	14,983	2280	13.2
Parental Psychiatric Diagnosis						
No	27,488	6780	19.8	29,536	7902	21.1
Yes	1454	343	19.1	1722	434	20.1
Urbanicity						
Capital	4410	782	15.1	4880	631	11.4
Capital Suburb	4404	908	17.1	4845	803	14.2
Provincial Cities	3324	837	20.1	3739	730	16.3
Provincial Towns	7616	2019	21.0	8404	2289	21.4
Rural Area	9188	2577	21.9	9390	3883	29.3
Parental Immigrant Status						
No	27,427	6621	19.4	29,095	7680	20.9
Yes	1515	502	24.9	2163	656	23.3
Maternal Pregnancy Smoking						
No	23,270	5541	19.2	24,895	6203	19.9
Yes	5672	1582	21.8	6363	2133	25.1

^a^Maternal analyses included 6706 cases and 29,359 controls

^b^Paternal analyses included 7647 cases and 31,947 controls

^c^Percent exposed

Mothers of cases were less likely to have been exposed (18.6% vs. 20.0%). We observed an inverse association between any maternal occupational asthmagen exposure and ASD in the children (adjusted OR (aOR): 0.92, 95% CI: 0.86–0.99) (Fig. 1). This inverse association was largely driven by the exposures to latex (aOR: 0.90, 95% CI: 0.82–0.98). There was no overall association between any low molecular weight maternal exposure and ASD (crude and adjusted associations in Additional file 1: Table S3). Results were almost identical in sensitivity analyses with additional adjustment for maternal asthma diagnosis prior to delivery (data not shown).

The paternal occupational asthmagen exposure prevalence in cases and controls was 20.4% and 21.2%, respectively. There was no association between any occupational asthmagen and ASD in the children (aOR: 0.98, 95% CI: 0.92–1.05) (Fig. 2). Paternal agricultural antigen exposure was inversely associated with ASD with some but not all of this association explained by confounding (cOR: 0.63, 95% CI: 0.54–0.73; aOR: 0.71, 95% CI: 0.61–0.84) (Additional file 1: Table S4). Some paternal occupational asthmagen categories were positively associated with ASD, including bioaerosols (aOR: 1.21, 95% CI: 1.03–1.41), pharmaceutical drugs (manufacturing or extensive handling) (aOR: 1.75, 95% CI: 1.03–2.97), and metals (aOR: 1.15, 95% CI: 1.02, 1.28). Additional adjustment for paternal asthma diagnosis prior to the child’s delivery did not impact the regression results (data not shown).

There was no association between strict childhood autistic disorder and either maternal or paternal occupational asthmagen exposure (maternal aOR: 0.92, 95% CI: 0.81–1.03; paternal aOR: 1.04, 95% CI: 0.94–1.16). Maternal asthmagen exposure was not associated with increased odds of ASD with intellectual disability (aOR: 1.10, 95% CI: 0.93–1.29), but odds of ASD with intellectual disability was elevated in paternal asthmagen exposed compared to unexposed (aOR: 1.19, 95% CI: 1.02–1.37).

In models that included an interaction term between maternal occupational asthmagen exposure and maternal asthma diagnosis prior to the child’s birth, we did not find evidence that asthma modified the association between maternal exposure and ASD (Table 2). The adjusted odds ratio between maternal occupational asthmagen exposure and ASD among mothers without an asthma diagnosis and mothers with an asthma diagnosis was 0.93 (95% CI: 0.86–0.99) and 0.73 (95% CI: 0.43–1.25), respectively. When we defined maternal asthma history as either a maternal asthma diagnosis or a dispensed asthma medication, we similarly did not see evidence of effect modification by maternal asthma history (data not shown). There was evidence of a multiplicative interaction between paternal occupational asthmagen exposure and paternal asthma diagnosis in relation to ASD (Table 2). The odds ratio for paternal occupational asthmagen exposure and ASD was 0.97 (95% CI: 0.90–1.03) among fathers without an asthma diagnosis, and 1.70 (95% CI: 1.12–2.58) among fathers with an asthma diagnosis.

**Table 2:** Independent and joint effects of parental occupational asthmagen exposure and parental asthma diagnosis^a^

		Control		ASD			
		N	%	N	%	aOR^bc^	95% CI
Maternal Asthmagen	Maternal Asthma						
No	No	23,053	78.5	5339	79.6	REF	.
Yes	No	5768	19.6	1226	18.3	0.93	0.86–0.99
No	Yes	430	1.5	120	1.8	1.24	1.00–1.54
Yes	Yes	108	0.4	21	0.3	0.90	0.55–1.47
Paternal Asthmagen	Paternal Asthma						
No	No	24,655	77.2	5975	78.1	REF	.
Yes	No	6655	20.8	1515	19.8	0.97	0.90–1.03
No	Yes	517	1.6	111	1.5	0.99	0.79–1.23
Yes	Yes	120	0.4	46	0.6	1.68	1.17–2.40

^a^Parental asthma defined as asthma diagnosed by a specialist prior to child’s birth

^b^Maternal model includes maternal asthmagen exposure, child’s year of birth, child’s sex, maternal age at birth, paternal age of birth, total income of parents, parity, highest parental education, history of parental psychiatric diagnosis prior to child’s date of birth, urbanicity of birth place, maternal immigrant status, maternal smoking, maternal asthma diagnosis by a specialist prior to child’s date of birth, and interaction term between maternal asthmagen exposure and maternal asthma diagnosis by a specialist prior to the child’s date of birth (6706 cases and 29,359 controls)

^c^Paternal model includes paternal asthmagen exposure, child’s year of birth, child’s sex, maternal age at birth, paternal age of birth, total income of parents, parity, highest parental education, history of parental psychiatric diagnosis prior to child’s date of birth, urbanicity of birth place, paternal immigrant status, maternal smoking, paternal asthma diagnosis by a specialist prior to child’s date of birth, and interaction term between paternal asthmagen exposure and paternal asthma diagnosis by a specialist prior to the child’s date of birth (7647 cases and 31,947 controls)

We did not see heterogeneity in the association between asthmagen exposure and ASD in models in which we included an interaction term with child sex. For maternal asthmagen exposures, the association with ASD was similar for male children (aOR: 0.93, 95% CI: 0.86–1.01) and female children (aOR: 0.89, 95% CI: 0.77–1.02) (p-value for interaction: 0.5). With paternal asthmagen exposures, we also did not see any heterogeneity of effect by sex (Among male children: aOR: 0.99, 95% CI: 0.92–1.07; Among female children: aOR: 0.94, 95% CI: 0.83–1.07; *p*-value for interaction: 0.5).

We did not see an association between parental asthma diagnosis prior to the birth of the child and ASD, but we did see a suggestion of association between parental asthma diagnosis after the birth of the child and ASD (data not shown).

**Fig. 3:**
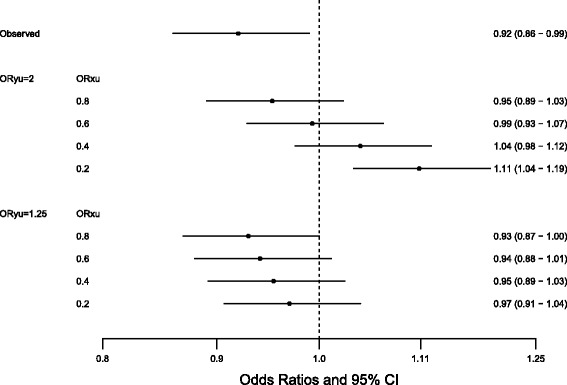
Sensitivity to unobserved confounder for the association between maternal occupational asthmagen exposure and ASD. Sensitivity analysis assumed fixed prevalence of unmeasured confounder among unexposed of 30%. ORyu = association between the unmeasured confounder and ASD. ORxu = association between the unmeasured confounder and maternal occupational asthmagen exposure

Our sensitivity analyses suggest that the inverse association between maternal exposure and ASD could be sensitive to an unobserved confounder (Fig. 3). Assuming that the prevalence of the unobserved confounder among the unexposed is 30%, a confounder that increases the odds of ASD by 1.25 times and decreases the odds of maternal occupational asthmagen exposure 0.6 times would bring the observed adjusted odds ratio of 0.92 (95% CI: 0.86–0.99) to the unmeasured confounder adjusted odds ratio to 0.94 (95% CI: 0.88–1.01) (Fig. 3). While adjustment for unmeasured confounding could bring the association to the null, the confounding would need to be implausibly strong to mask a positive association.

## Discussion

We observed an inverse association between any maternal exposure to asthmagens and ASD. This small protective association was largely explained by latex exposures. In contrast, for fathers the overall association between any workplace asthmagen exposure and ASD was null. Paternal exposure to agricultural antigens was inversely associated with ASD whereas there was some suggestion of weak positive associations between occupational exposures to bioaerosols, pharmaceutical drugs, and metals. Some of these associations could be chance findings resulting from multiple comparisons or could reflect bias, such as unmeasured confounding. Alternatively, these paternal associations may indicate a direct association between paternal occupational exposures and male reproductive health that is not mediated through the mother.

Inverse associations could be explained by health of the parent influencing both employment decisions and ASD risk in the children. Individuals with medical complications resulting from asthmagen exposures may be more likely to either change jobs or stop working, a concept known as the healthy worker effect [28]. The healthy worker effect has been observed in studies examining asthma occupational risk factors [29, 30]. The plausibility of the healthy worker effect explaining the inverse association may depend on the specific asthmagen. For example, it is possible that people with hypersensitivities to latex gloves may use non-latex gloves resulting in exposure misclassification. If such misclassification is differential by case status, then this could also potentially explain the inverse association. Differences in employment choices in cases compared to control parents that are not directly related to parental health conditions may also explain inverse associations.

Our approach was to focus specifically on occupational agents that cause asthma. Other air pollution and occupational epidemiology studies have examined a wide array of toxicants, some of which are asthmagens, in relation to ASD, though results were inconsistent [9–15, 31–39]. Examples of air pollutants that can induce or exacerbate asthma include certain metals (cadmium, chromium, cobalt, manganese, nickel), aldehydes, styrene and ethylene oxide [40]. While our results suggest a null association or weakly positive between parental occupational asthmagenic metal or metalworking fluid exposures and ASD, some studies report stronger positive associations with air pollutant exposures to metals [9, 12], including asthmagenic metals such as cadmium [9, 12], manganese [12], and nickel [9, 12]. Other studies suggested no association between hazardous air pollutant exposure to asthmagenic metals and ASD [10, 11], and no relation of occupational exposures to metals and ASD [13]. ASD has also been linked to acetaldehyde and formaldehydes [11], agents that could be classified as highly reactive agents, cleaning agents, or irritants in the asthma specific job exposure matrix [9–12]. We did not see any association between these specific asthmagen categories and ASD.

McCanlies et al. [13] did not see associations between parental occupational exposure to disinfectants and ASD, whereas another study reported an association between maternal occupational exposure to disinfectants and ASD [14]. We see a crude association between both maternal and paternal occupational exposure to cleaning and disinfectant products and ASD that was attenuated upon adjusting for confounders. In our previous study examining maternal asthmagen exposures in relation to ASD in the SEED case-control study, our result was consistent with the null though the odds ratio estimate was in the positive direction [16].

Asthma has a strong genetic component. In particular, gene-environment interactions have been reported between specific types of asthmagen exposures, single nucleotide polymorphisms, and occupational asthma [41, 42] and atopy is an established risk factor for asthma [43, 44]. Consequently, many individuals exposed to asthmagens may not mount a biological response. Thus, it may be difficult to identify links between asthmagens and outcomes without consideration of different susceptibilities to asthma. We also recognize that different asthmagenic agents trigger different biological response pathways. For example, HMW agents and some LMW agents can produce occupational asthma through an immunoglobulin-E (IgE) response whereas other LMW agents trigger occupational asthma through IgE-independent mechanisms.

We examined the interaction between parental occupational asthmagen exposures and parental asthma under the hypothesis that mothers with a history of asthma may be more likely to mount a biological immune response to asthmagens but saw no interaction. Intriguingly, the association between paternal occupational asthmagen exposure and ASD was stronger among fathers with asthma. This does not fit with the hypothesis of a maternal immune mechanism and may be a chance association since only 120 controls and 46 cases had exposed fathers with an asthma diagnosis. Alternatively, this association may result from unmeasured confounding, may reflect another mechanism by which asthmagens could impact paternal reproductive health, or may be result of multiple testing.

Although we adjusted for many possible confounders, our study is limited by the possibility of residual confounding, given that covariates were acquired from nationwide registries. In sensitivity analyses, we found that additional adjustment for parental asthma prior to the child’s birth did not impact the results. However, health conditions may be particularly challenging to account for because they are likely under-reported in the National Patient Register, which only includes diagnoses by a specialist. Our sensitivity analyses revealed that any unmeasured confounders, including confounding related to healthy worker effect, could account for the inverse association, but would need to be very strongly associated with exposure and outcome to mask a positive association between occupational asthmagens and ASD. We found differences in characteristics comparing parents missing occupational asthmagen exposure information to parents with enough information to estimate exposure. This may potentially limit the generalizability of the findings, though we did adjust for these characteristics in our analyses.

Occupational exposure assessment was also limited by data available in the Danish Registries. Companies supply job codes to Danish authorities for tax purpose and Statistics Denmark then validates and imputes other DISCO-88 codes based on data sources such as union membership records and educational registers [45]. While we incorporated industry into the exposure assessment, our ability to complete the expert assessment steps recommend by the asthma JEM authors was limited because we did not have information on job tasks. We were also unable to examine timing of exposure within pregnancy and did not include secondary job exposures. The asthma JEM assumes that all people with the same job and industry codes have the same binary value for exposure: it does not account for individual differences in exposure intensity and use of personal protective equipment. The JEM was designed to favor high specificity at the price of sensitivity [8] with prior information suggesting a specificity close to 1 and a sensitivity of 0.4 [46]. Despite these asthma JEM limitations, mothers and fathers with workplace asthmagen exposure during the pregnancy period were more likely to receive a diagnosis of asthma after the birth of the child than those without exposure (Mothers: 2.7% vs 1.9%; Fathers: 1.5% vs 1.1%). This suggests that the asthma JEM is measuring exposure well enough to capture an association with a known consequence of exposure.

Strengths of our study include large sample size, lack of potential for recall bias because of the use of administrative records, and minimal concern of selection bias related to control selection. The ASD cases were identified through ICD-10 codes in the Danish Psychiatric Central Register, which includes both inpatient and outpatient admissions for the period under study. Strict childhood autistic disorder diagnoses (F84.0) in the Danish Psychiatric Central Register have been validated [47]. There is some possibility of a lack of standard case assessment because the broader class of ASD diagnoses has not been validated. However, since Denmark has a national health system with free diagnostic assessments and interventions, we suspect that most ASD cases appear in the Register.

## Conclusions

In conclusion, this large population-based case-control study does not suggest a positive measurable association between parental occupational asthmagen exposure and ASD, despite previous reports of associations between air pollutants and other occupational exposures and ASD. Our sensitivity for unobserved confounders also suggests that healthy worker effect confounding would not be strong enough to bring the effect above the null. We found positive associations between paternal occupational exposures to bioaerosols, pharmaceutical drugs, and metals, though these could be chance findings resulting from multiple testing. We specifically examined asthmagen exposures, but future studies might harness the registers to examine other parental occupational patterns and exposures in relation to neurodevelopmental disorders. Future investigation of environmental risk factors for ASD may also benefit from consideration of genetic background and individual measurement of exposure biomarkers.

## Additional file

Additional file 1: Table S1. Parent and child characteristics by maternal occupational asthmagen exposure within 29,359 controls and 6706 cases. Table S2. Parent and child characteristics by paternal occupational asthmagen exposure within 31,947 controls and 7647 cases. Table S3. Associations between maternal occupational exposure to asthmagens during pregnancy and ASD (29,359 controls and 6706 ASD cases). Table S4. Associations between paternal occupational exposure to asthmagens during pregnancy and ASD (31,947 controls and 7647 ASD cases). (PDF 126 kb)
