# Supplementary material for: Parental exposures to occupational asthmagens and risk of autism spectrum disorder in a Danish population-based case-control study
Source: Environ Health. 2017 Mar 31;16:31. doi: 10.1186/s12940-017-0230-8 (PMC5374665; doi:10.1186/s12940-017-0230-8)
Supplement: Additional file 1: Table S1. — Parent and child characteristics by maternal occupational asthmagen exposure within 29,359 controls and 6706 cases. Table S2. Parent and child characteristics by paternal occupational asthmagen exposure within 31,947 controls and 7647 cases. Table S3. Associations between maternal occupational exposure to asthmagens during pregnancy and ASD (29,359 controls and 6706 ASD cases). Table S4. Associations between paternal occupational exposure to asthmagens during pregnancy and ASD (31,947 controls and 7647 ASD cases). (PDF 126 kb) [file 12940_2017_230_MOESM1_ESM.pdf]

Appendix. Parental exposures to occupational asthmagens and risk of autism spectrum disorder in a Danish population-based case-control study.

Supplemental Table 1: Parent and child characteristics by maternal occupational asthmagen exposure within 29,359 controls and 6,706 cases.

	Control			ASD		
	No	Yes		No	Yes	
	N	N	% [a]	N	N	% [a]
Child's Sex						
Male	12019	2946	19.7	4275	982	18.7
Female	11464	2930	20.4	1184	265	18.3
Child's Year of Birth						
1993-1997	7504	2428	24.4	2109	610	22.4
1998-2002	8200	1778	17.8	2262	407	15.2
2003-2007	7779	1670	17.7	1088	230	17.5
Parity						
1	11015	2551	18.8	2977	640	17.7
2	8868	2202	19.9	1828	411	18.4
≥3	3600	1123	23.8	654	196	23.1
Maternal Age at Child's Birth						
≤25 years	3325	940	22.0	910	240	20.9
26-30 years	9596	2466	20.4	2152	488	18.5
31-35 years	7801	1859	19.2	1696	373	18.0
≥36 years	2761	611	18.1	701	146	17.2
Total Parental Income						
<200,000 DKK	393	88	18.3	146	33	18.4
200,000-399,999 DKK	5522	1764	24.2	1666	463	21.7
400,000-599,999 DKK	11511	2877	20.0	2567	583	18.5
≥600,000 DKK	6057	1147	15.9	1080	168	13.5
Highest Parental Education						
Basic School	1119	448	28.6	387	132	25.4
Upper Secondary School	1085	175	13.9	309	60	16.3
Vocational School	10076	2509	19.9	2335	533	18.6
Higher Education	11203	2744	19.7	2428	522	17.7

Parental Psychiatric Diagnosis						
No	22444	5614	20.0	5044	1166	18.8
Yes	1039	262	20.1	415	81	16.3
Urbanicity						
Capital	3331	603	15.3	1079	179	14.2
Capital Suburb	3333	709	17.5	1071	199	15.7
Provincial Cities	2793	698	20.0	531	139	20.7
Provincial Towns	6276	1672	21.0	1340	347	20.6
Rural Area	7750	2194	22.1	1438	383	21.0
Maternal Immigrant Status						
No	22262	5460	19.7	5165	1161	18.4
Yes	1221	416	25.4	294	86	22.6
Maternal Pregnancy Smoking						
No	19128	4622	19.5	4142	919	18.2
Yes	4355	1254	22.4	1317	328	19.9

[a] Percent exposed.

Supplemental Table 2: Parent and child characteristics by paternal occupational asthmagen exposure within 31,947 controls and 7,647 cases.

	Control			ASD		
	No	Yes		No	Yes	
	N	N	% [a]	N	N	% [a]
Child's Sex						
Male	12888	3387	20.8	4778	1227	20.4
Female	12284	3388	21.6	1308	334	20.3
Child's Year of Birth						
1993-1997	8373	2744	24.7	2464	748	23.3
1998-2002	8673	2259	20.7	2416	579	19.3
2003-2007	8126	1772	17.9	1206	234	16.3
Parity						
1	11128	2805	20.1	3136	765	19.6
2	9855	2529	20.4	2127	541	20.3
≥3	4189	1441	25.6	823	255	23.7
Paternal Age at Child's Birth						
≤25 years	1871	779	29.4	535	199	27.1
26-30 years	7727	2275	22.7	1849	509	21.6
31-35 years	9214	2258	19.7	2013	512	20.3
≥36 years	6360	1463	18.7	1689	341	16.8
Total Parental Income						
<200,000 DKK	606	163	21.2	179	53	22.8
200,000-399,999 DKK	6459	2503	27.9	2020	714	26.1
400,000-599,999 DKK	11729	3216	21.5	2732	672	19.7
≥600,000 DKK	6378	893	12.3	1155	122	9.6
Highest Parental Education						
Basic School	1684	648	27.8	553	197	26.3
Upper Secondary School	1237	232	15.8	378	70	15.6
Vocational School	9981	4021	28.7	2442	888	26.7
Higher Education	12270	1874	13.2	2713	406	13.0

Parental Psychiatric Diagnosis						
No	23921	6459	21.3	5615	1443	20.4
Yes	1251	316	20.2	471	118	20.0
Urbanicity						
Capital	3646	460	11.2	1234	171	12.2
Capital Suburb	3687	626	14.5	1158	177	13.3
Provincial Cities	3120	600	16.1	619	130	17.4
Provincial Towns	6882	1848	21.2	1522	441	22.5
Rural Area	7837	3241	29.3	1553	642	29.2
Paternal Immigrant Status						
No	23393	6232	21.0	5702	1448	20.3
Yes	1779	543	23.4	384	113	22.7
Maternal Pregnancy Smoking						
No	20318	5115	20.1	4577	1088	19.2
Yes	4854	1660	25.5	1509	473	23.9

[a] Percent exposed.

Supplemental Table 3: Associations between maternal occupational exposure to asthmagens during pregnancy and ASD (29,359 controls and 6,706 ASD cases).

	Control Exposed	ASD Exposed	Crude		Adjusted	
	N	N	OR	95% CI[b]	OR	95% CI[b]
Any Asthmagen	5876	1247	0.91	0.85 - 0.98	0.92	0.86 - 0.99
Any HMW[c]	4219	879	0.90	0.83 - 0.97	0.93	0.86 - 1.01
Animals	258	58	0.98	0.74 - 1.31	1.00	0.74 - 1.35
Fish	124	33	1.17	0.79 - 1.71	0.98	0.65 - 1.47
Flour	70	19	1.19	0.72 - 1.98	1.18	0.69 - 2.01
Plants	10	4	1.76	0.55 - 5.59	1.49	0.45 - 4.94
Mites	16	5	1.37	0.50 - 3.74	1.42	0.50 - 4.03
Enzymes	47	15	1.40	0.78 - 2.50	1.38	0.75 - 2.55
Latex	3309	679	0.89	0.81 - 0.97	0.90	0.82 - 0.98
Bioaerosols	194	44	0.99	0.72 - 1.38	1.22	0.86 - 1.72
Drugs	393	79	0.88	0.69 - 1.12	1.01	0.79 - 1.31
Any LMW[d]	2970	709	1.05	0.96 - 1.15	0.99	0.90 - 1.08
Reactive	2271	508	0.98	0.88 - 1.08	0.93	0.83 - 1.03
Isocyanate	45	12	1.17	0.62 - 2.21	1.29	0.66 - 2.54
Cleaning	1834	488	1.18	1.06 - 1.31	1.04	0.93 - 1.16
Wood	44	11	1.10	0.57 - 2.12	1.26	0.63 - 2.52
Metals	335	80	1.05	0.82 - 1.34	1.14	0.89 - 1.48
Any Mixed	491	110	0.98	0.80 - 1.21	1.03	0.83 - 1.28
Metalworking	78	24	1.35	0.85 - 2.13	1.50	0.93 - 2.44
Textile	168	36	0.94	0.65 - 1.35	0.80	0.55 - 1.17
Agricultural	245	50	0.89	0.66 - 1.21	1.07	0.78 - 1.48
Irritant Peaks	69	11	0.70	0.37 - 1.32	0.75	0.39 - 1.45

[a] Adjusted for child's year of birth, child's sex, maternal age at birth, paternal age of birth, total income of parents, parity, highest parental education, history of parental psychiatric diagnosis prior to child's date of birth, urbanicity of birth place, maternal immigrant status, and maternal smoking. [b] 95% CI= 95% confidence interval. [c] HMW = High Molecular Weight. [d] LMW = Low Molecular Weight.

Supplemental Table 4: Associations between paternal occupational exposure to asthmagens during pregnancy and ASD (31,947 controls and 7,647 ASD cases).

	Control Exposed	ASD Exposed	Crude		Adjusted	
	N	N	OR	95% CI[b]	OR	95% CI[b]
Any Asthmagen	6775	1561	0.95	0.90 - 1.01	0.98	0.92 - 1.05
Any HMW[c]	1986	535	1.13	1.03 - 1.25	1.14	1.02 - 1.26
Animals	647	135	0.87	0.72 - 1.05	0.96	0.79 - 1.17
Fish	162	47	1.21	0.88 - 1.68	1.20	0.85 - 1.68
Flour	152	38	1.05	0.73 - 1.49	1.03	0.71 - 1.50
Plants	26	7	1.13	0.49 - 2.60	1.25	0.52 - 2.98
Mites	9	0	.	.	.	.
Enzymes	124	29	0.98	0.65 - 1.47	0.99	0.65 - 1.51
Latex	367	117	1.34	1.08 - 1.65	1.12	0.90 - 1.40
Bioaerosols	835	226	1.13	0.98 - 1.32	1.21	1.03 - 1.41
Drugs	54	21	1.63	0.98 - 2.70	1.75	1.03 - 2.97
Any LMW[d]	4249	1063	1.05	0.98 - 1.13	1.04	0.96 - 1.12
Reactive	949	251	1.11	0.96 - 1.28	1.01	0.87 - 1.18
Isocyanate	522	121	0.97	0.79 - 1.18	0.97	0.78 - 1.19
Cleaning	554	162	1.23	1.03 - 1.46	1.04	0.86 - 1.25
Wood	1130	241	0.89	0.77 - 1.02	0.92	0.79 - 1.07
Metals	1685	450	1.12	1.01 - 1.25	1.15	1.02 - 1.28
Any Mixed	2327	477	0.85	0.76 - 0.94	0.90	0.81 - 1.00
Metalworking	919	254	1.16	1.01 - 1.34	1.14	0.98 - 1.33
Textile	89	22	1.03	0.65 - 1.65	0.90	0.56 - 1.46
Agricultural	1319	201	0.63	0.54 - 0.73	0.71	0.61 - 0.84
Irritant Peaks	808	201	1.04	0.89 - 1.22	1.11	0.94 - 1.30

[a] Adjusted for child's year of birth, child's sex, maternal age at birth, paternal age of birth, total income of parents, parity, highest parental education, history of parental psychiatric diagnosis prior to child's date of birth, urbanicity of birth place, paternal immigrant status, and maternal smoking. [b] 95% CI= 95% confidence interval. [c] HMW = High Molecular Weight. [d] LMW = Low Molecular Weight.
